# A case of CDKL5 deficiency disorder with a novel intragenic multi-exonic duplication

**DOI:** 10.1038/s41439-024-00296-7

**Published:** 2024-11-08

**Authors:** Takato Akiba, Shino Shimada, Katsumi Imai, Satoru Takahashi

**Affiliations:** 1 NHO Shizuoka Institute of Epilepsy and Neurological Disorders, National Epilepsy Center, Shizuoka, Japan; 2 Pediatrics and Adolescent Medicine, Juntendo University, Graduate School of Medicine, Tokyo, Japan; 3 Department of Clinical Genetics, Juntendo University, Graduate School of Medicine, Tokyo, Japan; 4 Department of Pediatrics, Asahikawa Medical University, Asahikawa, Japan

**Keywords:** Epilepsy, Genetics of the nervous system

## Abstract

We present a case of suspected CDKL5 deficiency disorder (CDD) in which a novel intragenic multi-exonic duplication in the *CDKL5* gene was identified using next-generation sequencing and multiple ligation-dependent probe amplification. This duplication was assumed to result in a shift of the reading frame and the introduction of a premature stop codon. This case highlights the importance of careful phenotyping and comprehensive genetic testing to detect rare structural variants in CDD patients.

Cyclin-dependent kinase-like 5 (*CDKL5*) deficiency disorder (CDD; OMIM 300672), also known as early infantile epileptic encephalopathy 2, is characterized by severe early-onset drug-resistant epilepsy with motor, cognitive, visual, and autonomic disturbances resulting from nonfunctional or absent CDKL5 protein^1^. The prevalence of CDD is estimated at 1 in 40,000–60,000 live births^2,3^. The *CDKL5* gene is located at Xp22.1, and females are more commonly affected than males because of the lethality of germline mutations in most males during fetal development. To date, more than 250 pathogenic *CDKL5* variants have been reported; point mutations account for more than half of these variants, with only a few reported intragenic duplications, and detailed clinical information is rarely reported^4^. Here, we report a novel intragenic multi-exonic duplication in a patient with CDD.

An 11-year-old girl was referred to the NHO Shizuoka Institute of Epilepsy and Neurological Disorders. Born at 36 weeks with a birth weight of 2794 g, she was the first child of a nonconsanguineous Japanese couple. Her mother had a history of self-limited epilepsy with centrotemporal spikes (SeLECTS). The neonatal period of the patient was unremarkable; however, at the age of 2 months, she experienced symmetrical bilateral tonic seizures accompanied by upward rolling of the eyeballs and eyelid myoclonia. She developed epileptic spasms (ESs) with series formation at 3 months of age. No dysmorphic features or abnormal laboratory findings were observed. The patient was diagnosed with infantile epileptic spasms syndrome on the basis of ictal electroencephalogram (EEG) findings and hypsarrhythmia. Neither antiepileptic drugs (valproic acid, vitamin B6, zonisamide, levetiracetam, lamotrigine, nitrazepam, and vigabatrin) nor adrenocorticotropic hormone therapy controlled her seizures. At the age of 11 years, ESs and brief tonic seizures persisted. In addition to hypsarrhythmia, interictal EEG worsened, with a low-amplitude basic rhythm and multifocal spikes mixed in irregular high-amplitude slow waves, similar to a suppression-burst pattern. Ketone diet therapy was difficult to implement due to frequent vomiting. Head magnetic resonance imaging revealed mild atrophy of the left hemisphere. Brain positron emission tomography revealed mild hyperintensity from the left parietal to temporal regions. No structural brain abnormalities requiring surgical treatment were found. She is currently bedridden and requires assistance in all aspects of daily living. She has not achieved vision pursuit or neck control and has no language understanding. Based on female sex and early-onset epileptic encephalopathy, we suspected CDD.

After comprehensive counseling, we explained the risks and benefits of genetic testing. This study was approved by the Ethics Review Committee of our institution, and we obtained written informed consent from the patient’s parents for whole-exome sequencing using genomic DNA extracted from the patient’s peripheral lymphocytes. Targeted next-generation sequencing of *SLC9A6*, *TCF4*, *MBD5*, *MECP2*, *CDKL5*, and *FOXG1* using the hybrid capture method was performed at the Kazusa DNA Research Institute, Chiba, Japan, a previously undocumented heterologous large-scale duplication in the *CDKL5* gene (NM_003159) was identified. To determine the region of the duplication, multiple ligation-dependent probe amplification (MLPA) of *CDKL5* was performed using the SALSA MLPA Probemix P189-B1 CDKL5 (MRC Holland, Amsterdam, the Netherlands), which confirmed the duplication of exons 9–16 (Fig. 1A). To identify the structural variation, we performed RT‒PCR using RNA derived from peripheral white blood cells. The forward primers targeted exons 6 and 14, and the reverse primers targeted exons 10 and 18 (Fig. 1B and Supplementary Table 1). Using the exon 14 forward primer and exon 10 reverse primer, a cDNA amplification band was observed only in the patient sample. Sequencing analysis of the patient sample confirmed that the duplication of exons 9–16 was intragenic and occurred in tandem (Fig. 1B). The combined use of exon 6F and exon 10R primers for RT–PCR should result in a 2055 bp band in addition to the 423 bp band observed in this case. However, the longer band was not detected because PCR was performed using the standard method instead of long and accurate PCR. This duplication was assumed to result in a shift of the reading frame and the introduction of a premature stop codon (NP_001310218.1: p.Val793Argfs*20). This heterozygous, large, tandem duplication in *CDKL5* was absent from controls in public databases, such as the dbSNP (http://www.ncbi.nlm.nih.gov/snp/) and Genome Aggregation Database (gnomAD, https://gnomad.broadinstitute.org/). The variation was also not registered in ClinVar (https://www.ncbi.nlm.nih.gov/clinvar/) or the Human Gene Mutation Database (https://www.hgmd.cf.ac.uk/ac/index.php). Because low-level mosaicism of *CDKL5* variants is sufficient to develop drug-resistant epilepsy^5^, the carrier status of the parents was not analyzed in this study. We thought that the mother, who has self-limited epilepsy, may not be a carrier of the *CDKL5* variant. This duplication was assumed to have arisen de novo and to alter the protein length, corresponding to PM4 and PM6, according to the American College of Medical Genetics and Genomics criteria^6^. Therefore, this duplication was considered likely pathogenic (PM2, PM4, PM6, PP4).

**Fig. 1:**
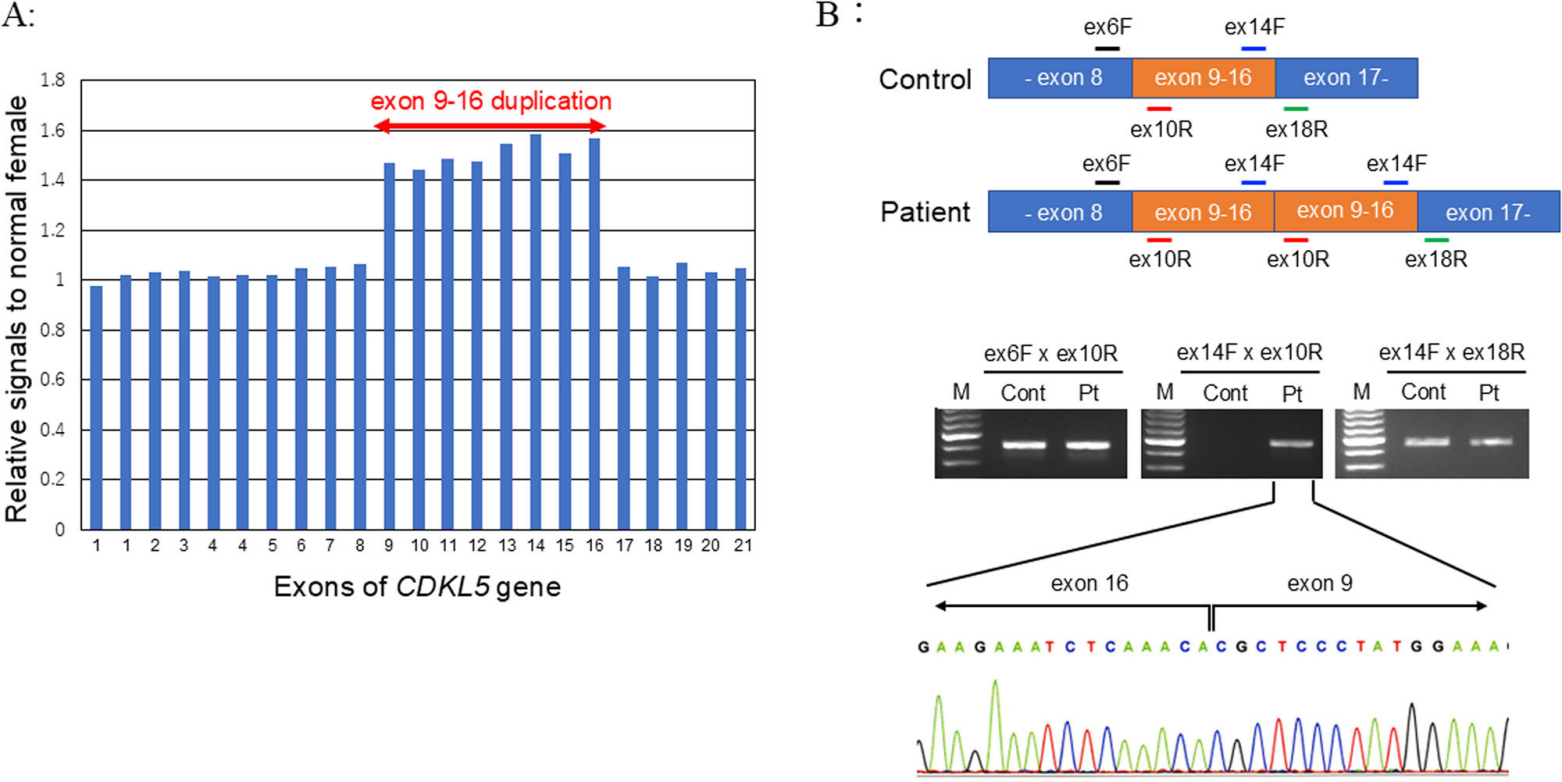
Details of the genetic analysis results. **A** Duplication in *CDKL5* exons 9–16 detected by multiple ligation-dependent probe amplification. **B** Using forward and reverse primers, we detected tandem duplication of CDKL5 exons 9–16. We also confirmed that the end of exon 16 and the beginning of exon 9 were connected without any deletion. ex, exon; F, forward primer; R, reverse primer.

The patient presented a typical CDD phenotype, and the duplication mutation seemed to disrupt the catalytic domain function of the protein. The genotype‒phenotype correlation in CDD is unclear. While some reports have shown that the severity of manifestations in heterozygous females and hemizygous males is equivalent^7^, several studies have reported sex-based phenotypic differences, with males having a higher frequency of ESs and brain atrophy, and females often presenting with atypical Rett syndrome in addition to early-onset developmental and epileptic encephalopathy^8^.

Patients with mutations within the catalytic domain and frameshift mutations at the end of the C-terminal region reportedly exhibit more severe phenotypes^9^. However, we previously reported no difference in seizure prognosis or developmental outcomes between gene mutations occurring in the catalytic domain and those occurring in its downstream region^10^. It is difficult to adequately determine the phenotypic severity based only on the mutation location and sex.

Reports of CDD due to exon duplication in *CDKL5* are limited due to clinical genetic testing capabilities^11–14^. In our case, the use of careful phenotyping and next-generation sequencing with MLPA detected a disease-causing duplication variant in *CDKL5*, confirming tandem duplication. Next-generation sequencing and exon-level array comparative genomic hybridization have been reported to achieve a high diagnostic rate^2^. However, exon duplications can be missed by the typical analysis methods of clinical practice alone. Recently, de novo duplication of an exon leading to a frameshift was identified by long-read genome sequencing and whole-genome sequencing (WGS)^13,14^. Long-read genome sequencing identified a de novo L1-mediated insertion in *CDKL5*, resulting in the duplication of exon 3^13^. WGS detected a 63 kb de novo duplication, resulting in tandem duplication of exons 5–15, which was confirmed to be a stop-gain variant by cDNA sequencing^14^. Recent advances in genetic analysis techniques may result in the identification of more cases with exon duplication in *CDKL5*.

We report a case of CDD with a novel intragenic multi-exonic duplication. The clinical course of this patient is the most severe phenotype of CDD reported to date. Although CDD with identified exon duplications is extremely rare, the possibility should be considered when performing genetic analysis. This work was partly supported by the MHLW Research Program on rare and intractable diseases (grant number JPMH23FC1013).

## HGV database

The relevant data from this Data Report are hosted at the Human Genome Variation Database at 10.6084/m9.figshare.hgv.3447.

## Supplementary information

Supplementary Table 1
